# Supplementary material for: Production and Health Management from Grazing to Confinement Systems of Largest Dairy Bovine Farms in Azores: A Farmers’ Perspective
Source: Animals (Basel). 2021 Nov 27;11(12):3394. doi: 10.3390/ani11123394 (PMC8697991; doi:10.3390/ani11123394)
Supplement: Supplementary file 1 [file animals-11-03394-s001.zip › animals-1453406-supplementary.pdf]

animals

Table S1. Characterization of farms and main biosecurity measures in dairy farms with fixed (FMP) versus mobile (MMS) milking machines.

Factor	FMP Farms	MMS Farms	*p* value
Characterization of farms			
Animals (estimated numbers by producers at survey time):			
Total	213.1±11.7 (60-520) [1)]	157.8±21.9 (52-400)	0.02
Adult dairy cows (>24 months)	108.8±5.8 (30-300)	82.0±8.2 (33-150)	0.05
Heifers (12–24 months)	42.8±3.2 (7-150)	30.7±4.9 (8-80)	0.05
Calves and heifers (<12 months)	39.7±2.8 (6-133)	33.8± 6.3 (9-100)	0.20
Males (calf's and bulls from 100 farms)	11.4±1.8 (0-90)	17.0±6.4 (1-100)	0.10
Culling (from 11 farms)	18.0±4.6 (0-40)	12.7±2.3 (8-15)	0.70
Housing- Animals exclusively confined:			
Adult cows (>24 months)	24.1% (21/87) [2)]	5.6% (1/18)	0.08
Dry cows	14.9% (13/87)	11.1% (2/18)	0.66
Heifers (12–24 months) (*n* = 104)	7.0% (6/86)	5.6% (1/18)	0.82
Calves (<12 months)	74.1% (65/87)	55.6% (10/18)	0.10
Production System:			
Dual purpose (milk and meat)	24.1% (21/87)	50.0% (9/18)	0.03
Semi-intensive production system*	72.4% (63/87)	83.3% (15/18)	0.33
Organic dairy production**	2.3% (2/87)	0.0% (0/18)	0.51
Part of an animal welfare program	44.8% (39/87)	16.7% (3/18)	0.03
Refrigerated milk bulk tank	90.8% (79/87)	22.2% (4/18)	<0.001
Biosecurity measures			
Isolated box/local for sick animals	58.6% (50/87)	27.8% (5/18)	0.02
Animal sales to other farms	27.6% (24/87)	27.8% (5/18)	0.98
Animal purchase from other farms	24.1% (21/87)	27.8% (5/18)	0.75
Quarantine of purchased animals (from 26 farms)	38.1% (8/21)	40.0% (2/5)	0.93
Bury dead animals on pasturage lands	98.9% (86/87)	100% (18/18)	0.65
Give farm clotting to veterinary assistance	0.0% (0/87)	0.0% (0/18)	1.00
Animal transhumance (public roads)	42.5% (37/87)	83.3% (15/18)	0.002
Other animal species in the farm (dogs, cats, goats, sheep, swine, horses)	54.0% (47/87)	50.0% (9/18)	0.76

n: number of respondents. %: percentage of farms with an affirmative response. [1)] arithmetic mean ± standard error of mean (min - max). [2)] (*n*/N): number of affirmative response/number of total respondents. * Semi-intensive production system: the cows stay outdour, using grazing system, in the great part of the day. ** Organic dairy production: defined by the Council Regulation (EC) No 834/2007 of 28 June 2007 (https://eur-lex.europa.eu/legal-content/EN/TXT/?uri=CELEX%3A32007R0834, accessed on 11 11 2026).

Table S2. Calving, rearing management and facilities in dairy farms with fixed (FMP) versus mobile (MMS) milking systems.

Factor	FMP Farms	MMS Farms	*p* value
Calving			
Number of calving's	137.4±7.8 (25-380) [1)]	92.9±12.6 (25-200)	0.007
Seasonal calving according to owner grass availability	8.1% (7/87)	22.2% (4/18)	0.07
Avoid summer calving (*n* = 104)	20.7% (18/87) [2)]	29.4% (5/17)	0.43
Calving pens	51.7% (45/87)	11.1% (2/18)	0.002
Age at first calving (in Months.)	26.7±0.3 (22-30)	25.5±0.4 (24-30)	0.19
Estimated calving interval (in days) (*n* = 102)	399±1.2 (370-450)	395±3.8 (380-430)	0.15
At least one Cesarean section in farm (*n* = 104)	30.2% (26/86)	16.7% (3/18)	0.24
Retained placenta as a problem	25.3% (22/87)	27.8% (5/18)	0.83
Placenta elimination (*n* = 104):			
Put in the trash (*n* = 6)	7.0% (6/86) [a]	0.0% (0/18)	
Buried at pasture (*n* = 63)	64.0% (55/86) [b]	44.4% (8/18) [a]	0.04
Put on septic pit (*n* = 14)	14.0% (12/86) [a]	11.1%(2/18) [b]	
Not ruled out (*n* = 21)	15.0% (13/86) [a]	44.4%(8/18) [a]	
Calves			
Calves management:			
Calf barns	89.7% (78/87)	72.2% (8/13)	0.05
Regular disinfection of the calf shed (*n* = 91)	88.5% (69/78)	84.6% (11/13)	0.69
Colostrum to calves (days)	4.7±0.3 (1,8)	3.9±0.5 (1-7)	0.10
Colostrum banking	17.2% (15/87)	11.1% (1/18)	0.52
Dead calves up to 28 days [3)]	1.8±0.1 (1-5)	1.9±0.2 (1-5)	0.45
Calves diarrhea as main problem	70.1% (61/87)	70.6% (12/18)	0.77
Calves pneumonia as main problem	34.5% (30/87)	72.2% (13/18)	0.003
Self-reared calves for beef	28.7% (25/87)	61.1% (11/18)	0.008
Age at weaning (in Mo.)	3.0±0.1 (2-8)	3.0±0.2 (2-6)	0.88
Addition of concentrate supplementation in diet	97.7% (85/87)	88.9% (16/18)	0.08
Water availability *ad libitum*	100% (87/87)	100% (18/18)	1.00
Calves preventive measures:			
Calves´ vaccination up to 12 old's week	13.8% (12/87)	0.0% (0/18)	0.09
Vaccination of pregnant cattle to prevent neonatal diarrhea and pneumonia	25.3% (22/87)	5.6% (1/18)	0.07
Calves deworming	98.9% (86/87)	100% (18/18)	0.65
Vitamins supplementation	89.7% (78/87)	88.9% (18/18)	0.92
Disbudding (<2 Months of age) / Dehorning methods (*n* = 104):			
Thermal / Hot-iron disbudding (*n* = 71)	68.6 (59/86)% [a]	66.7% (12/18) [a]	
Chemical / Caustic disbudding (*n* = 16)	17.4%(15/86) [b]	5.6% (1/18) [b]	
Scissor disbudding (*n* = 15)	11.6% (10/86) [b]	27.6% (5/18) [c]	0.21
Wire/Saw Dehorning (*n* = 2)	2.3% (2/86)	0% (0/18)	

n: number of respondents. %: percentage of farms with an affirmative response. [a,b,c] Different superscript letters for the same column: p <0.05. [1)] arithmetic mean ± standard error of mean (min - max). [2)] (*n*/N): number of affirmative response/number of total respondants. [3)] Scale 1 to 5, according to the number of death calves up to 28 days: 1:0-5; 2:5-10; 3:10-15; 4:15-20; 5:>20.

Table S3. Lameness and lameness control in dairy farms with fixed (FMP) versus mobile (MMS) milking systems.

Factor	FMS Farms	MMS Farms	*p* value
Lameness:			
Lameness is a major problem in my farm	41.4% (36/87) [1)]	38.9% (7/18)	0.85
Lameness program implemented	34.5% (30/87)	16.7% (3/18)	0.14
Trimming:			
By specialized technician	95.4% (83/87)	77.8% (14/18)	0.01
When lameness incidence peaks (n = 101):	74.1% (63/85)	93.8% (15/16)	0.09
Score number of trimming frequency (n = 102) [2)]	2.5±0.1 (1-5)	2.0±0.3 (1-4)	0.08
Footbaths:			
Footbath implementation	48.3% (42/87)	5.6% (1/18)	0.001
Footbath change weekly frequency or less (n = 39)	76.3% (29/38)	100% (1/1)	0.58
Genetic selection for lameness reduction	75.9% (66/87)	55.6% (10/18)	0.08

%: percentage of farms with an affirmative response. n: number of respondents. [1)] (n/N): number of affirmative response/number of total respondents. [2)] Scale 1 to 5, according to the percentage of trimmed cows during 2020: 1:10%; 2:10-20%; 3:20-30%; 4:30-40%; 5: >40%.

Table S4. Dry-off time and prepartum measures adopted in dairy farms with fixed (FMP) versus mobile (MMS) milking systems.

Factor	FMP Farms	MMS Farms	*p* value
Drying-off system			
Drying bolus (micronutrients)	27.6% (24/87) [1)]	22.2% (4/18)	0.63
Sealant application	19.5% (17/87)	5.6% (1/18)	0.15
Dry-off antibiotherapy	95.4% (83/87)	88.9% (16/18)	0.28
Parenteral selenium administration	23.0% (20/87)	16.7% (3/18)	0.56
Score of dry period length [3)]	3.1±0.1 (1-4) [2)]	3.1±0.2 (1-4)	0.84
Prepartum care			
Calcium bolus (last week)	17.2% (15/87)	5.6% (1/18)	0.21
Glucose IV (at calving)	3.4% (3/87)	5.6% (1/18)	0.67
Calcium IV (at calving)	11.5% (10/87)	16.7% (3/18)	0.44
Monensin (at calving)	27.6% (24/87)	27.8% (5/18)	0.98
Injectable multi-vitamin supplement (last two weeks)	11.5% (10/87)	16.7% (3/18)	0.54
Injectable vitamin D3 (8-10 days prepartum)	6.9% (6/87)	11.1% (2/18)	0.53

[1)] (n/N): number of affirmative response/number of total respondents. [2)]) arithmetic mean ± standard error of mean (min - max). [3)] Scale 1 to 4, according to the most prevalent length of the dry period: 1- <30 days; 2-30 to 45 days; 3- 45 to 60 days; 4- >60 days. IV: intravenous route.
